# Supplementary figures and images for: SipD and IpaD induce a cross-protection against Shigella and Salmonella infections
Source: PLoS Negl Trop Dis. 2020 May 28;14(5):e0008326. doi: 10.1371/journal.pntd.0008326 (PMC7282677; doi:10.1371/journal.pntd.0008326)

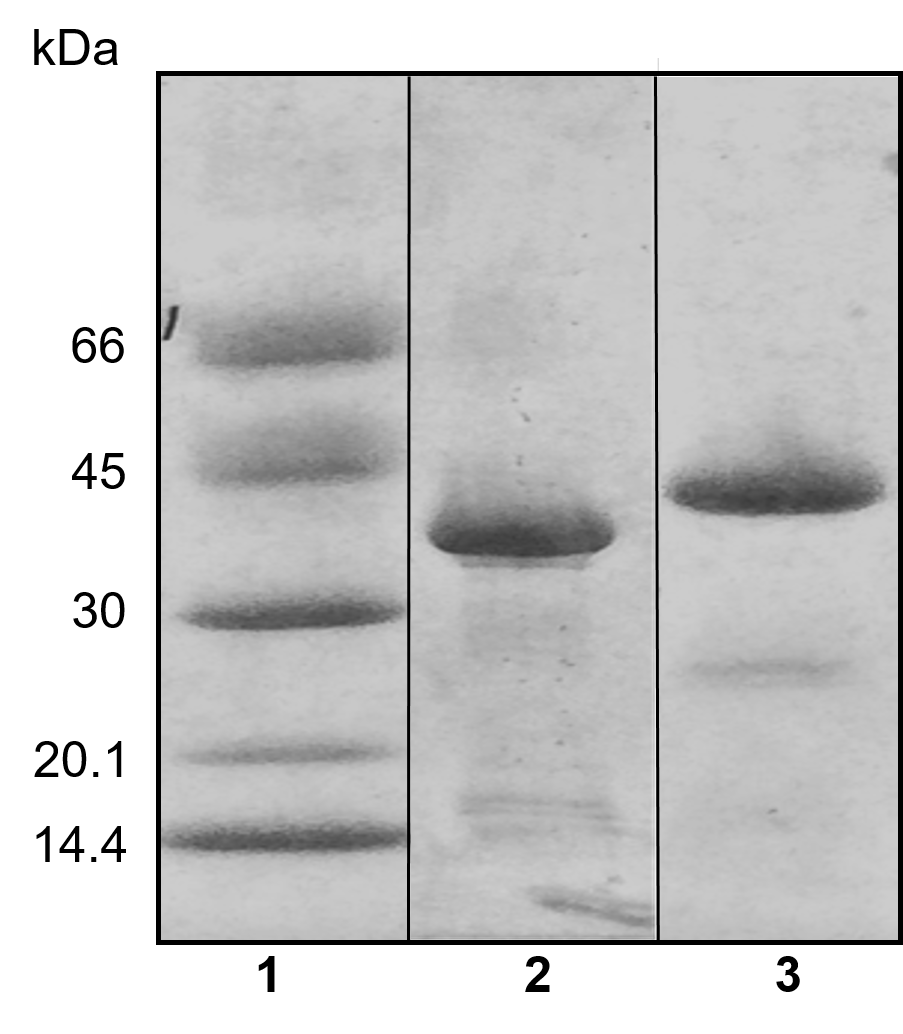

Supplement: S1 Fig — SDS-PAGE / Coomassie blue staining (reducing conditions) of purified recombinant proteins. PolyHis-IpaD (37.1 kDa, lane 2) and polyHis-SipD (38.2 kDa, lane 3) are shown with molecular mass markers in kilodaltons (kDa) (lane 1). (TIF) [file pntd.0008326.s001.tif]

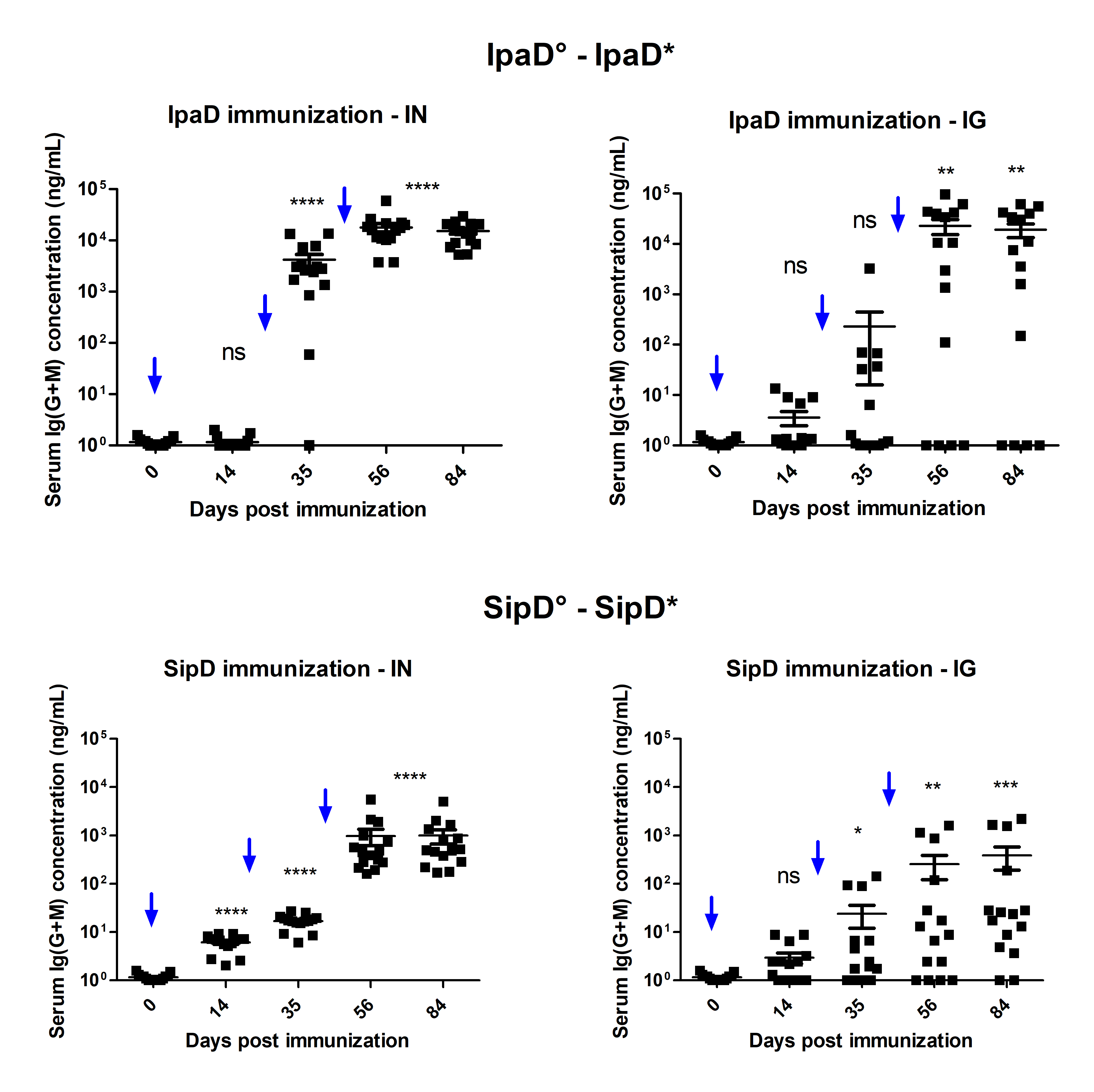

Supplement: S2 Fig — Mice were immunized three times (time indicated with arrows) with IpaD (A) or SipD (B) by the IN route (left panels) or IG route (right panels) as described in Materials and Methods. Homologous responses of Ig(G+M) antibodies specific for IpaD or SipD were quantified by sandwich ELISA. Data represent mean concentrations (ng/mL) and the standard errors (SEM) from 14–16 individual mice per group. (**** p < 0.0001, *** 0.0001 < p < 0.001, ** 0.001 < p < 0.01 and * 0.01 < p < 0.1. ns: non significant) comparing the antibody responses on days post-immunization versus those on day 0 (nonparametric Mann-Whitney test).°: indicates injected immunogen; *: indicates biotinylated recombinant protein. (TIF) [file pntd.0008326.s002.tif]

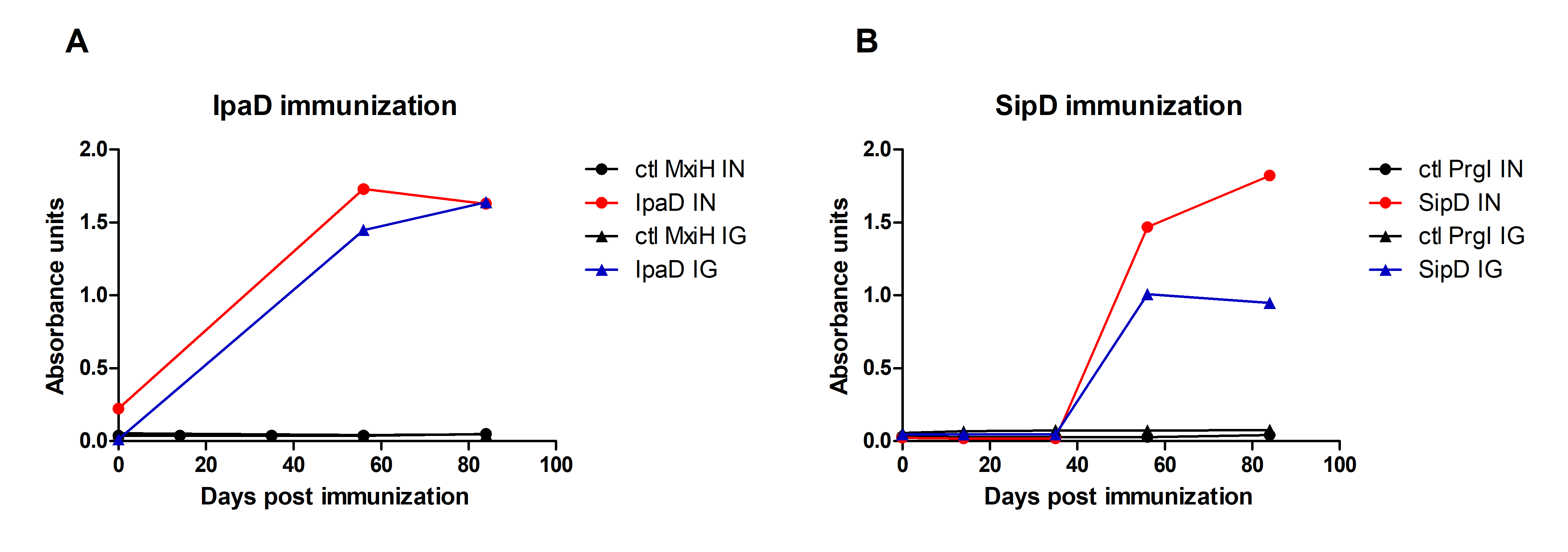

Supplement: S3 Fig — Mice were immunized three times intranasally (IN) or intragastrically (IG) with IpaD (A) or SipD (B) as described in Materials and Methods. Example of specificity of Ig(G+M) responses is shown for one mouse per route of immunization, and was assessed by using biotinylated unrelated recombinant proteins, sharing the same His-tag as IpaD and SipD at their C-terminus. Control (ctl) His-tagged MxiH (needle protein of Shigella injectisome) or His-tagged PrgI (needle protein of Salmonella injectisome) were used for mice immunized with IpaD and SipD respectively and quantified by sandwich ELISA. Data represent absorbance units obtained with sera of mice diluted 1000 fold. (TIF) [file pntd.0008326.s003.tif]

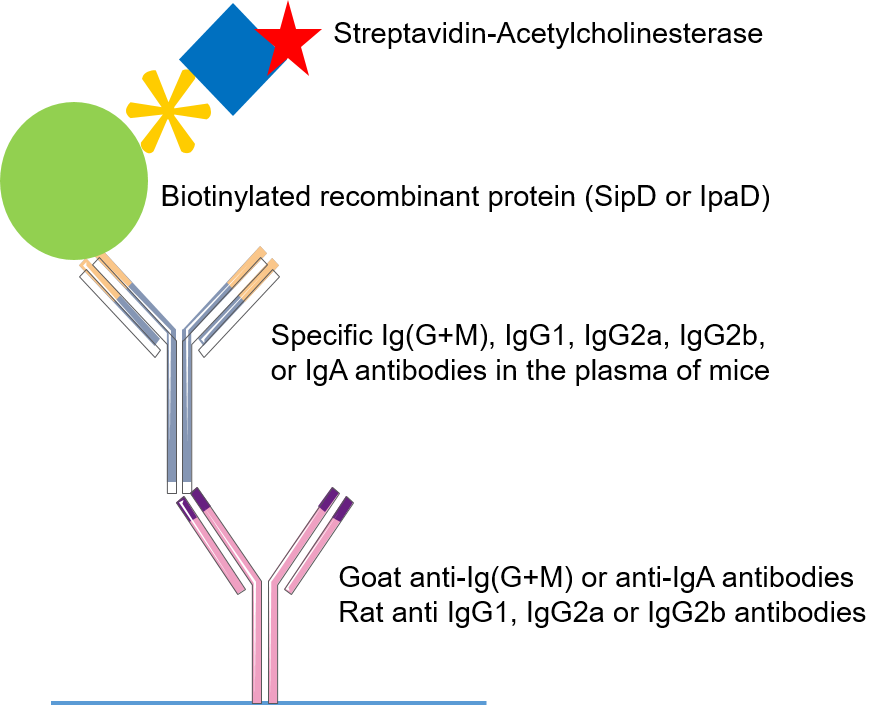

Supplement: S4 Fig — A sandwich ELISA test was performed to measure the concentrations of circulating antibodies (immune response after immunizations (Ig(G+M), IgG1, IgG2a, IgG2b and IgA, see experimental procedures) (TIF) [file pntd.0008326.s004.tif]

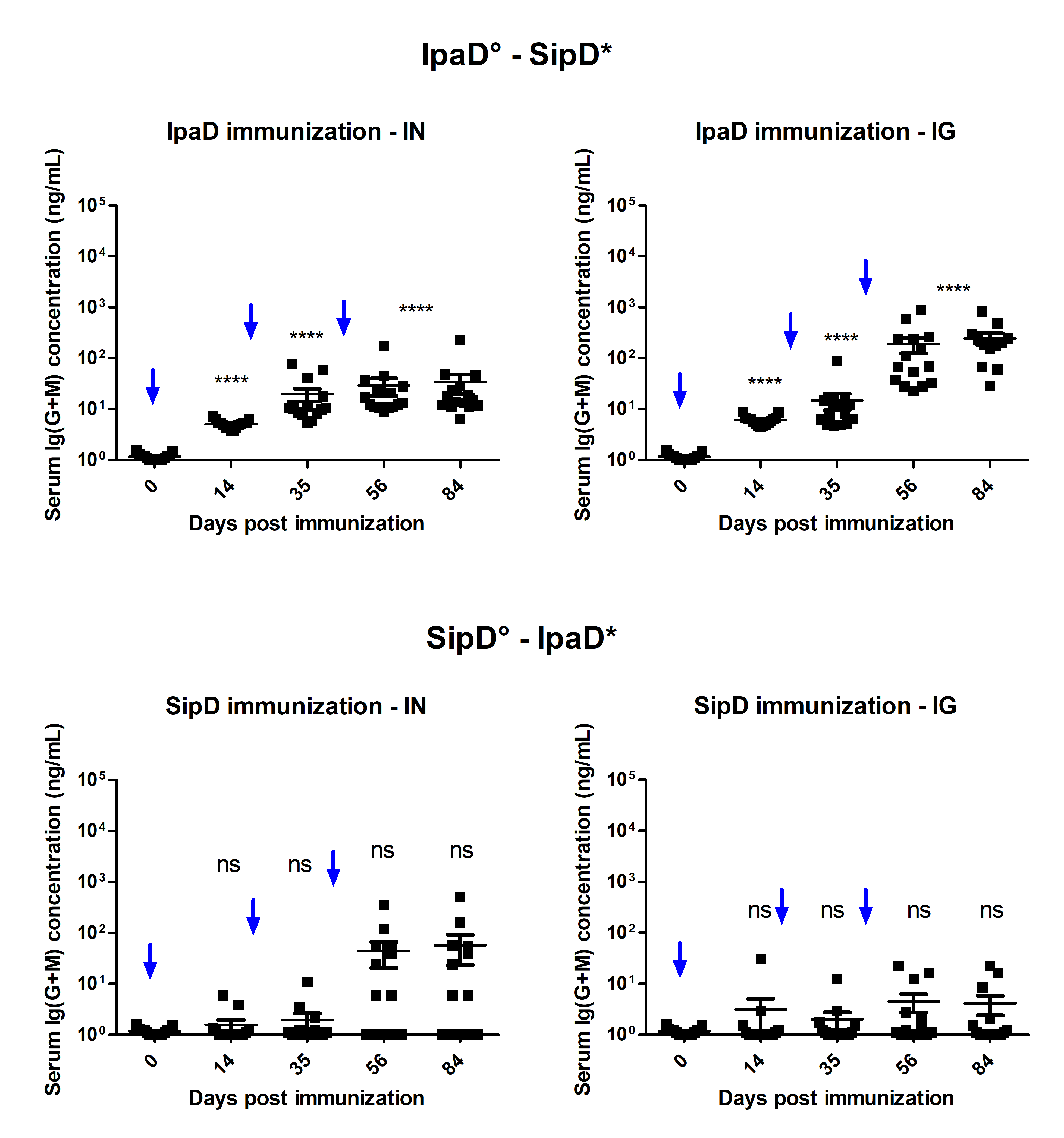

Supplement: S5 Fig — Mice were immunized three times (time indicated with arrows) with IpaD (A) or SipD (B) by the IN route (left panels) or IG route (right panels) as described in Materials and Methods. Heterologous responses of Ig(G+M) antibodies specific for SipD (from mice immunized with IpaD) or SipD (from mice immunized with IpaD) were quantified by sandwich ELISA. Data represent mean concentrations (ng/mL) and the standard errors (SEM) from 14–16 individual mice per group. (**** p < 0.0001, *** 0.0001 < p < 0.001, ** 0.001 < p < 0.01 and * 0.01 < p < 0.1. ns: non significant) comparing the antibody responses on days post-immunization versus those on day 0 (nonparametric Mann-Whitney test).°: indicates injected immunogen; *: indicates biotinylated recombinant protein. (TIF) [file pntd.0008326.s005.tif]

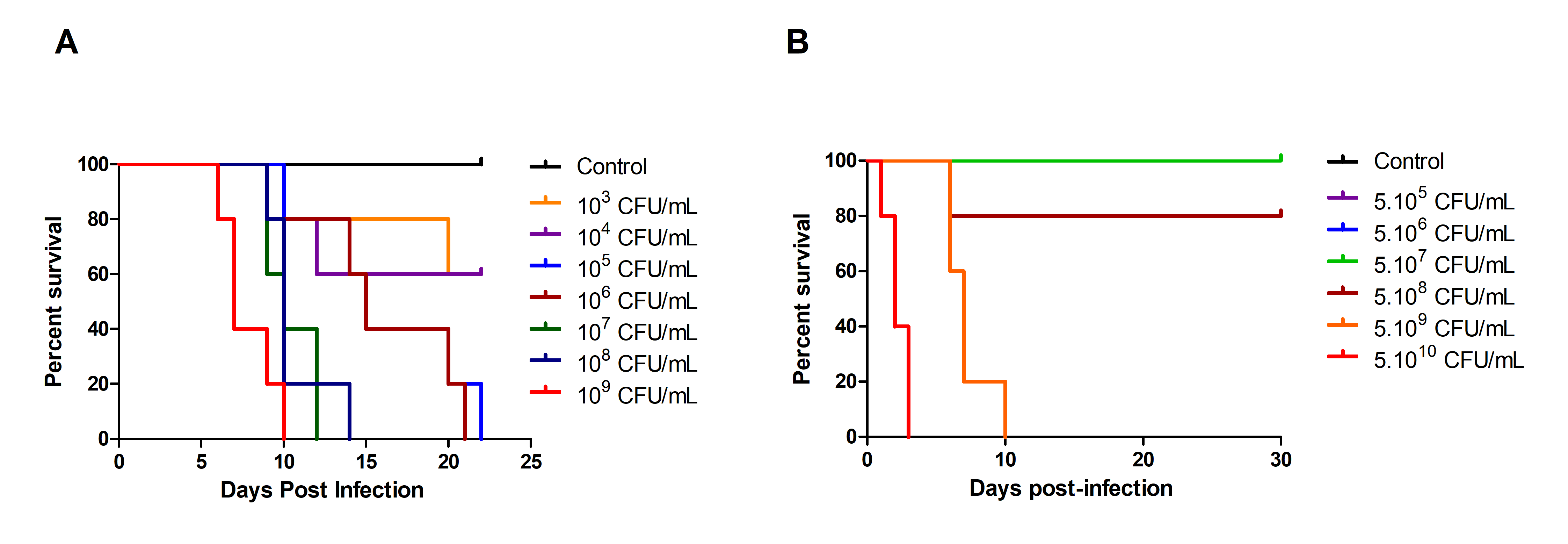

Supplement: S6 Fig — Serial dilutions of S. Typhimurium (from 2.102 to 2.108 CFU) and S. flexneri 2a (5.105 to 5.1010 CFU) were administered intragastrically (S. Typhimurium) or intranasally (S. flexneri 2a) to 20- to 22-week-old female BALB/c mice (5 mice per group). The 50% mouse lethal dose (LD 50) was calculated by the method of Reed and Muench. (TIF) [file pntd.0008326.s006.tif]

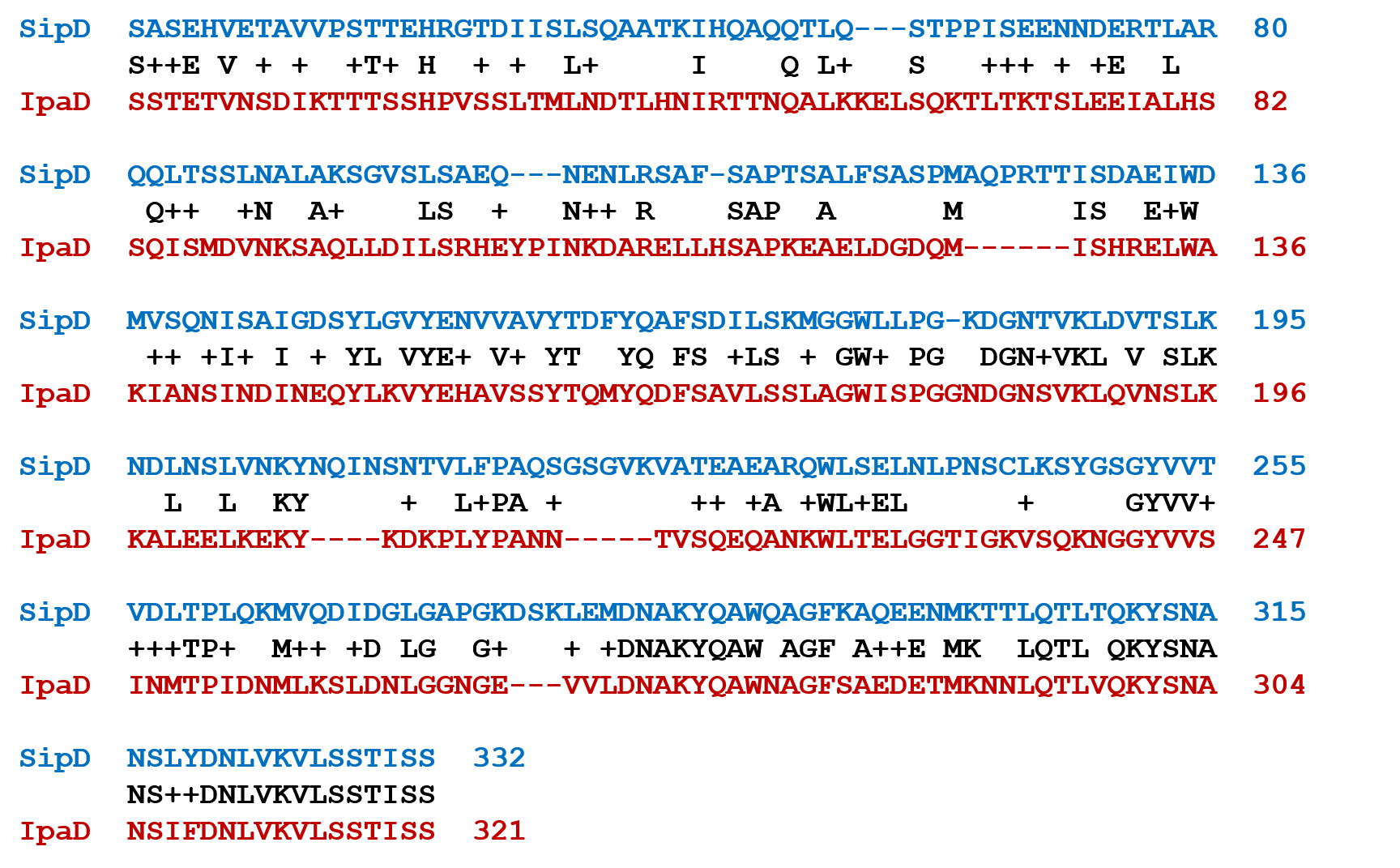

Supplement: S7 Fig — Alignment sequences of IpaD from S. flexneri 2a (accession number SVF87366.1) and SipD from S. Typhimurium (accession number AAA86617.1) were performed using BLAST (Basic local alignment search tool) from NCBI (https://blast.ncbi.nlm.nih.gov/). SipD sequence is represented in blue and IpaD sequence in red. Identical aminoacids are represented in black and similar aminoacids by a “+”. Sequence identity is 38.17%. (TIF) [file pntd.0008326.s007.tif]
