# Supplementary material for: SipD and IpaD induce a cross-protection against Shigella and Salmonella infections
Source: PLoS Negl Trop Dis. 2020 May 28;14(5):e0008326. doi: 10.1371/journal.pntd.0008326 (PMC7282677; doi:10.1371/journal.pntd.0008326)
Supplement: Attachment — Submitted filename: responses to reviewers complete.docx [file pntd.0008326.s010.docx]

**Dear Editor,**

**In the first part of this document I have answered to all the comments that were in the main body of your mail. As some of other comments and requests were also present in the attached file you sent to me (and not in the main body of the mail), I decided to join in the second part of this document the answers to the attached word document.**

Reviewer #1: The methods that are described are sufficient but it is unclear why the authors chose to measure Ig concentrations in ng/ml rather than the typical titers. Also, no discussion of PrgI production is presented making that protein irrelevant.
The LD50 for Shigella is very, very high. There is no discussion of differential colony staining on Congo red. It is likely that the vast majority of colonies are white producing an artificially high LD50. At these challenge doses all the mice should be dead in 24 hr.

Reviewer #2: Methods have been done and presented properly.

Reviewer #3: This is a well written manuscript giving a reasoned account of how the authors selected the antigens to induce a cross-protection against Shigella and Salmonella infections in a mouse model. The experiment was well designed and the homologous and heterologous antibody responses following the selected antigens immunization makes this work interesting.

Reviewer #4: As seen in the comments to the authors, the following concerns are noted for the methods:
- the analyses used histidine-tagged proteins or biotinylated the tagged proteins for the ELISA analyses
- no direct measure for T cell-specific responses.
- the LD50 for the Shigella infection resulted in an 80% survival and required a very high infectious dose for the challenge studies.

--------------------

**Results**


-Does the analysis presented match the analysis plan?

-Are the results clearly and completely presented?

-Are the figures (Tables, Images) of sufficient quality for clarity?


Reviewer #1: The results are presented but for most of the manuscript are overstated. It is hard to the manuscript seriously when IpaD does not provide protection after IN vaccination and IN challenge. That should have been the positive control. Again, with such a high dose of Shigella, it is unclear how any of the mice could have survived. Like the shigella are "white" and are missing the T3SS.
The authors need to decide on OG vs IG. This is OG ORO-gastric

Reviewer #2: Like Methods, results have been done and presented properly. However, since the authors purified the recombinant proteins, I think it was better if they share some photos of the recombinant proteins.

Reviewer #3: The results are completely presented. However, the quality and resolution for Figures 1- 6 are not satisfied.

Reviewer #4: The analyses match the plan and the figures/tables are presented clearly. Please note the minor concerns to improve the clarity of the figure legends.

--------------------

**Conclusions**


-Are the conclusions supported by the data presented?

-Are the limitations of analysis clearly described?

-Do the authors discuss how these data can be helpful to advance our understanding of the topic under study?

-Is public health relevance addressed?


Reviewer #1: The conclusions are NOT supported by the results. Many of the conclusions drawn by the authors lack significance - especially in the challenge experiments.

Reviewer #2: (No Response)

Reviewer #3: Conclusion is well-defined and supports the data presented. It is helpful to advance our understanding of the topic under study.

Reviewer #4: Based on the concerns noted in the methods and in the comments to the authors, some of the conclusions are not supported by the data presented. In particular, the cross-protection, efficacy in the Shigella challenge model, and the T helper cellular responses are not supported. The limitations to these are not clearly described.

--------------------

**Editorial and Data Presentation Modifications?**


Use this section for editorial suggestions as well as relatively minor modifications of existing data that would enhance clarity. If the only modifications needed are minor and/or editorial, you may wish to recommend “Minor Revision” or “Accept”.

Reviewer #1: (No Response)

Reviewer #2: There were some little writing mistakes that I should mention to be corrected:
-In the first line of the abstract please write food- and water-borne pathogens
-In line 37, please write emergence of multi drug resistances.
-In the lane 74, what is 30?
-Please italicize family, genus, species and subspecies all over the manuscript. (Line 48, for example).
-Please capitalize the word "Gram" in line 79.
-In Lane 135, please write "phosphate-buffered saline".
-I think there should be a space between the numbers (4, 37, etc.) and ºC. Please correct it all over the text.
-In line 142, there should be a space between g and for.
-In line 143 and 144, please write 2 × 102 to 2 × 108 CFU and 5 × 105 to 5 × 1010 CFU.
-All over the text you have used capital L for showing the volume, but somewhere, mainly in "Enzyme immunoassays" part in Materials and Methods section. Please correct this.
-In line 167, there should be a space between 10 and μg/mL.

Reviewer #3: The manuscript is recommended for publication once the minor revision is addressed.

Reviewer #4: (No Response)

--------------------

**Summary and General Comments**


Use this section to provide overall comments, discuss strengths/weaknesses of the study, novelty, significance, general execution and scholarship. You may also include additional comments for the author, including concerns about dual publication, research ethics, or publication ethics. If requesting major revision, please articulate the new experiments that are needed.

Reviewer #1: (No Response)

Reviewer #2: I read and enjoyed the present work. The main Idea was novel, rationale and interesting, experiments were designed appropriately and the results were presented and discussed professionally. I congratulate the authors for doing this study. However, there were some minor points that I'll mention in the following.
1. Since the authors purified the recombinant proteins, I think it was better if they share some photos of the recombinant proteins.
2. The authors have done IgG isotyping, however, they did not discuss the results of this experiment in "Discussion" part.
3. Somewhere in the manuscript, mainly from line 394 to the end of the "Discussion", no reference has mentioned, though it is necessary to refer to the previous studies. For example, i) Salmonella has two type three secretion systems involved in the pathogenicity [Reference is needed], ii) pathogenicity mechanisms are different between Salmonella and Shigella and particularly in regard to the involvement of the innate and adaptative immune response, iii) a more preponderant role of the intracellular cycle of Salmonella compared to Shigella [Reference is needed] and iv) a faster systemic dissemination of Salmonella in the murine model [Reference is needed]...
Finally, in my point of view this work deserves publishing in that esteemed journal.
Best,

Reviewer #3: The manuscript is recommended for publication once the minor revision is addressed.

Reviewer #4: The goal of the manuscript was to examine the immunogenicity and protective efficacy of the homologous type-III secretion system tip proteins IpaD from Shigella flexneri and SipD from Salmonella following intranasal and intragastric administration in a mouse model. Using various ELISA assays to measure antibody responses as well as performing lethal challenge studies in mice, the authors were able to detect IgG and IgA-specific responses and demonstrated that mice immunized with the proteins protected against each pathogen. More importantly, the authors provide evidence that the proteins can provide cross-protection against the other pathogen, which has important implications for vaccine development. Throughout the study, the authors perform robust analyses with a significant number of mice per treatment group, in which mice were immunized on days 0, 21, and 42 and challenged studies were performed on day 84. Results of the antibody responses in both the figures and supplemental figures were provided for both endpoint titers and throughout the course of the immunizations. In all, the data provide promising results for the pre-clinical efficacy analysis of the antibodies generated and the protection of mice that results from immunization of IpaD and SipD proteins. However, review of the manuscript identified some major and minor concerns that are highlighted below.

Major:
1. The authors state that the recombinant IpaD and SipD proteins were purified by cloning a poly-histidine tag on the 3’ end of the gene sequence. Moreover, the labeling of the recombinant proteins with biotin for the ELISAs appears to have the histidine tag intact for this procedure. There are no histidine-only controls provided in the analyses to determine if the antibodies generated are specific to the histidine tag. These controls are not only important for determining IpaD- or SipD-specific antibody responses, but are also important when evaluating the cross-protection analysis performed in the study. It is possible that the cross-protection is due to some or all the histidine tag present on the recombinant proteins and not due to the 38% identity between the two proteins.

2. The authors utilize IgG ratio analyses (Figure 4) to determine if the results are indicative of a humoral or cellular immune response of T helper cells. Direct measures and identification of specific T cell responses were not performed, which would strengthen the data. In the absence of such analyses, the author should state any conclusion from the ratio analyses as a hypothesis.

3. While the challenge studies use 100 LD50, there is concern that the LD50 dose used by the authors for S. flexneri resulted in 80% survival and not 50% survival (see supplemental figure 5). As the authors point out, the challenge dose of 5.1 x 10^10 is very high, but justify this high dose by pointing to a Shigella study in 2012 in which high LD50s were also used. This discussion is misleading, especially since the referenced study used doses of 6 x 10^7 and 1.3 x 10^8 for S. flexneri. There is a significant concern that the protection observed in the manuscript is not as promising given the dosages and model used. Furthermore, the concern is raised even more given the fact that the infection model is pulmonary-based and not physiologically relevant to Shigella infection. Animal models for Shigella can be difficult and vaccine development has not been successful despite promising animal model analyses throughout the literature. In the absence of repeating the analyses with better infection results as performed by other studies, the authors should better highlight and discuss the shortcomings from their analyses.


Minor:
1. The figure legends should define what the controls were for the analyses. It is assumed that the controls were mice treated with adjuvant + PBS, but the clarity would be helpful. It should also be stated in the Materials and Methods how many mice were used for the control groups. 15 is assumed but clarification would be helpful.

2. The figure legends should better clarify the immunogen and recombinant protein sentences (e.g. for Fig 1, lines 216-217). Suggestion is “…indicates biotinylated recombinant protein used for the ELISA analyses.”

3. The previous publication by the group in 2016 examined the secondary structure of recombinant SipD and PrgI using Far-UV CD spectroscopy. It is not clear why this analysis was not performed for IpaD.

**RESPONSES TO REVIEWERS (answers to the comments sent in the main body of the mail)**

**We thank reviewers for having reviewed carefully our publication and for all their comments, which allowed us to improve the manuscript. We have answered the best we could to the different points. We hope that reviewers will find it satisfactory for publication.**

Methods

Reviewer #1: The methods that are described are sufficient but it is unclear why the authors chose to measure Ig concentrations in ng/ml rather than the typical titers. Also, no discussion of PrgI production is presented making that protein irrelevant.

**Usually authors do not have the opportunity/standards to measure the absolute concentrations of the antibodies. As we had this possibility (using antibodies as standards), we prefered doing this, giving a precise measure of concentration, except for IgA, for which we did not have an IgA standard for the standard curve. Regarding PrgI, it was a mistake, and it has been corrected (suppression from line 280)**

The LD50 for Shigella is very, very high. There is no discussion of differential colony staining on Congo red. It is likely that the vast majority of colonies are white producing an artificially high LD50. At these challenge doses all the mice should be dead in 24 hr.

**We did perform a red selection of *S. flexneri 2a* colony. We have modified the manuscript consequently and added in the experimental procedures (line 118) « Bacteria were first grown at 37 °C on agar plates (trypticase soy (TCS) containing 0.01% Congo red (Serva) *for S. flexneri 2a* and LB plates *for S.* Typhimurium). For infection, a colony (Congo red-positive for *S. flexneri* 2a) was picked for a 5ml overnight (O/N) culture at 37 °C in LB medium, followed by a culture in the same medium with 1:100 of the first culture for 2 h under the same conditions.**

**The laboratory mouse is the animal of choice for most experimental medical research. Unfortunately, it is well-known that its utilization for shigellosis is inadequate for colonization of the intestinal tract and to elicit disease by this route. The lethality of Shigella strains administered intranasally has been found to correlate with virulence in humans (Voino-Yasenetsky, M. V., et al. 1962. Experimental pneumonia caused by bacteria of the Shigella group. Acta Morphol. Acad. Sci. Hung. 11:439–454). However, although far from perfect, the pulmonary based mouse model for shigellosis is the one used by the scientific community working on shigella vaccine. In the publication of Van de Verg LL, et al (Infection and Immunity, 1995), 10^6^ cfu of *S. flexneri 2a* induced 10% of mortality in Balb/C mice and 10^7^ cfu were necessary to have 2.2LD50. For our experiments we needed 10^7^ cfu/mouse (5.10^8^ cfu/mL) to obtain the LD50, which is in agreement with this publication. In an article from the laboratory who generously gave us the strain used (Phalipon et al, J. Exp. Med, 1995), it is mentioned (in the experimental procedures section) that 20 µL of a 5X10^8^ cfu/mL (=10^7^ cfu) administered intranasally corresponded to 10 fold less than the LD50. This reference has been added in the text (line 166).**

Reviewer #2: Methods have been done and presented properly.

**We thank the reviewer for this comment.**

Reviewer #3: This is a well written manuscript giving a reasoned account of how the authors selected the antigens to induce a cross-protection against Shigella and Salmonella infections in a mouse model. The experiment was well designed and the homologous and heterologous antibody responses following the selected antigens immunization makes this work interesting.

**We thank the reviewer for this comment.**

Reviewer #4: As seen in the comments to the authors, the following concerns are noted for the methods:

- the analyses used histidine-tagged proteins or biotinylated the tagged proteins for the ELISA analyses

- no direct measure for T cell-specific responses.

**Regarding His tagged proteins, we have added a supplementary figure that shows that the immune response is specific of the protein and not of the his tag (see supplementary figure 3).**

**Indeed we did not perform T cell-specific responses, and a sentence has been added in the result section line 323 « However this result should be taken with caution as it has not been confirmed by measuring directly the T cell specific response, as well as in the discussion section « All antibody subclasses are involved in the humoral response for both SipD and IpaD, regardless of route of immunization, and this highlights the importance of humoral (and particularly mucosal) immunity in protecting against *Salmonella* and *Shigella* infection. It has to be noted that protection against *Shigella* infection is better when mice are immunized by the IG route compared to the IN route. This might be correlated to the IgG subtype measurement balance in favor of a cellular response for IpaD by the IG route suggesting a significant contribution of the Th1 response for protection after IN challenge. It has to be noted that this hypothesis has not been verified by a direct measurement of the T cell specific response. »**

- the LD50 for the Shigella infection resulted in an 80% survival and required a very high infectious dose for the challenge studies.

**The LD50 of S. flexneri 2a has been calculated (reed and Muench method) at** **10^7^ cfu/mouse (5.10^8^ cfu/mL), and we agree that, at this concentration, we obtained 80% mice survival instead of 50%. However this dose is in agreement with the literature (Van de Verg LL, et al, Infection and Immunity, 1995, Martinez-Becerra FJ, et al, 2012) and 10 fold superior to what was obtained by Phalipon et al, 1995, who gave us the strain. We also have detailed our answer below, in the « overall comment » section.**

--------------------

Results

-Does the analysis presented match the analysis plan?

-Are the results clearly and completely presented?

-Are the figures (Tables, Images) of sufficient quality for clarity?

Reviewer #1: The results are presented but for most of the manuscript are overstated. It is hard to the manuscript seriously when IpaD does not provide protection after IN vaccination and IN challenge. That should have been the positive control. Again, with such a high dose of Shigella, it is unclear how any of the mice could have survived. Like the shigella are "white" and are missing the T3SS.

**IpaD gave a good protection by intragastric immunization (more than 60%) and a weak (non significant because of no delay in the mortality but still more than 20% of survival) protection by intranasal immunization. From our point of view, protection induced by IpaD administered intragastrically is also a good control. As the main difference that can explain partly this result is the balance IgG (2a+2b) vs IgG1, in favor of IgG (2a+2b) for IG immunization with IpaD, we can hypothesize on a specific role of Th1 response in protection. As no T cell response has been measured, it remains only an hypothesis. A sentence has been added in the result section line 323 « However this result should be taken with caution as it has not been confirmed by measuring directly the T cell specific response »** **as well as a pragraph in the discussion line 495 « All antibody subclasses are involved in the humoral response for both SipD and IpaD, regardless of route of immunization, and this highlights the importance of humoral (and particularly mucosal) immunity in protecting against *Salmonella* and *Shigella* infection. It has to be noted that protection against *Shigella* infection is better when mice are immunized by the IG route compared to the IN route. This might be correlated to the IgG subtype measurement balance in favor of a cellular response for IpaD by the IG route suggesting a significant contribution of the Th1 response for protection after IN challenge. It has to be noted that this hypothesis has not been verified by a direct measurement of the T cell specific response.»**

**LD50 is in agreement with the literature (Van de Verg LL, et al, Infection and Immunity, 1995, Martin-Becerra, 2012) and 10 fold superior to what was obtained by Phalipon et al, 1995, who gave us the strain.**

**A protocol of selection of red colonies was performed before the pre-culture and was not mentioned in the submission of the manuscript. This has been added in the experimental procedure section (line 118).**

The authors need to decide on OG vs IG. This is OG ORO-gastric

**If the reviewer agrees, we prefer to write IG instead of OG. The reason is because we have used a gastric cannula that goes into the esophagus for the administration of the bacteria. We have changed consequently the manuscript.**

Reviewer #2: Like Methods, results have been done and presented properly. However, since the authors purified the recombinant proteins, I think it was better if they share some photos of the recombinant proteins.

**I am sorry if I don’t understand well the question. We already have shown the SDS PAGE electrophoresis in supplementary S1 figure. Except that, we do not have the possibility to perform any electron microscopy. Regarding the circular dichroism (CD), it has been performed for SipD for the first article of B. Jneid (B. Jneid et al, 2016) and at this time IpaD was not produced. It has not been done after. We don’t have any more protein that was used for the immunizations. Thus, it does not seem relevant to reproduce now the protein to perform the CD, as it won’t be the same batch.**

Reviewer #3: The results are completely presented. However, the quality and resolution for Figures 1- 6 are not satisfied.

**We have increased the quality of the figures. I hope that it will appear satisfactory in the pdf version sent for the revision.**

Reviewer #4: The analyses match the plan and the figures/tables are presented clearly. Please note the minor concerns to improve the clarity of the figure legends.

**We thank the reviewer and have done all requested modifications.**

--------------------

Conclusions

-Are the conclusions supported by the data presented?

-Are the limitations of analysis clearly described?

-Do the authors discuss how these data can be helpful to advance our understanding of the topic under study?

-Is public health relevance addressed?

Reviewer #1: The conclusions are NOT supported by the results. Many of the conclusions drawn by the authors lack significance - especially in the challenge experiments.

**We hope that the answers we have provided and the modifications for improvement we have done will help to change the reviewer's mind.**

Reviewer #2: (No Response)

Reviewer #3: Conclusion is well-defined and supports the data presented. It is helpful to advance our understanding of the topic under study.

**We are grateful to the reviewer for its positive recommendation.**

Reviewer #4: Based on the concerns noted in the methods and in the comments to the authors, some of the conclusions are not supported by the data presented. In particular, the cross-protection, efficacy in the Shigella challenge model, and the T helper cellular responses are not supported. The limitations to these are not clearly described.

**We hope that the answers we have provided and the modifications for improvement we have done will help to change the reviewer's mind.**

--------------------

Editorial and Data Presentation Modifications?

Use this section for editorial suggestions as well as relatively minor modifications of existing data that would enhance clarity. If the only modifications needed are minor and/or editorial, you may wish to recommend “Minor Revision” or “Accept”.

Reviewer #1: (No Response)

Reviewer #2: There were some little writing mistakes that I should mention to be corrected:

-In the first line of the abstract please write food- and water-borne pathogens

**It has been corrected**

-In line 37, please write emergence of multi drug resistances.

**Corrected. “new” has been removed.**

-In the lane 74, what is 30?

**This was a mistake of bibliography formatting. It has been corrected and the corresponding reference has been added: Galan JE (2001) Salmonella interactions with host cells: type III secretion at work. *Annu. Rev. Cell Dev. Biol.* 17: 53–86**

-Please italicize family, genus, species and subspecies all over the manuscript. (Line 48, for example).

**It has been corrected for *Shigella flexneri*. According to the nomenclature (which has evolved with time), *Salmonella enterica* subsp *enterica* serovar Typhimurium should be italicized for *Salmonella* and *enterica*, but should be written as it (Typhimurium, not italicized, with T in capital). To clarify: 1- in the abstract (lines 23-24) “*Salmonella* Typhimurium” has been replaced by “*Salmonella* *enterica* serovar Typhimurium (*S.* Typhimurium)”, 2- line 29 *Salmonella* Typhimurium has been replaced by *S.* Typhimurium, 3- lines 65-66 S. Typhi has been replaced by *S. enterica* serovar Typhi.**

-Please capitalize the word "Gram" in line 79.

**Corrected, lines 49 and 87**

-In Lane 135, please write "phosphate-buffered saline".

**corrected**

-I think there should be a space between the numbers (4, 37, etc.) and ºC. Please correct it all over the text.

**corrected**

-In line 142, there should be a space between g and for.

**corrected**

-In line 143 and 144, please write 2 × 102 to 2 × 108 CFU and 5 × 105 to 5 × 1010 CFU.

**corrected**

-All over the text you have used capital L for showing the volume, but somewhere, mainly in "Enzyme immunoassays" part in Materials and Methods section. Please correct this.

**Corrected all along the text**

-In line 167, there should be a space between 10 and μg/mL.

**corrected**

Reviewer #3: The manuscript is recommended for publication once the minor revision is addressed.

**We are grateful to the reviewer for its positive recommendation.**

Reviewer #4: (No Response)

Use this section to provide overall comments, discuss strengths/weaknesses of the study, novelty, significance, general execution and scholarship. You may also include additional comments for the author, including concerns about dual publication, research ethics, or publication ethics. If requesting major revision, please articulate the new experiments that are needed.

Reviewer #1: (No Response)

Reviewer #2: I read and enjoyed the present work. The main Idea was novel, rationale and interesting, experiments were designed appropriately and the results were presented and discussed professionally. I congratulate the authors for doing this study.

**I gratefully thank the reviewer for its very positive comment and will do my best to answer all the mentioned points carefully.**

However, there were some minor points that I'll mention in the following.

1. Since the authors purified the recombinant proteins, I think it was better if they share some photos of the recombinant proteins.

**I am sorry if I don’t understand well the question. We already have shown the SDS PAGE electrophoresis in supplementary S1 figure. Except that, we do not have the possibility to perform any electron microscopy. Regarding the circular dichroism (CD), it has been performed for SipD for the first article of B. Jneid (B. Jneid et al, 2016) and at this time IpaD was not produced. It has not been done after. We don’t have any more protein that was used for the immunizations. Thus, it does not seem relevant to reproduce now the protein to perform the CD, as it won’t be the same batch.**

2. The authors have done IgG isotyping, however, they did not discuss the results of this experiment in "Discussion" part.

**We have added a pragraph in the discussion line 495 « All antibody subclasses are involved in the humoral response for both SipD and IpaD, regardless of route of immunization, and this highlights the importance of humoral (and particularly mucosal) immunity in protecting against *Salmonella* and *Shigella* infection. It has to be noted that protection against *Shigella* infection is better when mice are immunized by the IG route compared to the IN route. This might be correlated to the IgG subtype measurement balance in favor of a cellular response for IpaD by the IG route suggesting a significant contribution of the Th1 response for protection after IN challenge. It has to be noted that this hypothesis has not been verified by a direct measurement of the T cell specific response.»**

3. Somewhere in the manuscript, mainly from line 394 to the end of the "Discussion", no reference has mentioned, though it is necessary to refer to the previous studies. For example, i) Salmonella has two type three secretion systems involved in the pathogenicity [Reference is needed], ii) pathogenicity mechanisms are different between Salmonella and Shigella and particularly in regard to the involvement of the innate and adaptative immune response, iii) a more preponderant role of the intracellular cycle of Salmonella compared to Shigella [Reference is needed] and iv) a faster systemic dissemination of Salmonella in the murine model [Reference is needed]...

**References have been inserted to support these hypotheses: for i) ref 69 has been added, for iii) ref 70 and 71 have been added.**

**69 : Silva-Herzog E, Detweiler CS. Salmonella enterica replication in hemophagocytic macrophages requires two type three secretion systems. Infect Immun. 2010;78: 3369–3377. doi:10.1128/IAI.00292-10**

**70: Vazquez-Torres A, Fang FC. Cellular routes of invasion by enteropathogens. Curr Opin Microbiol. 2000;3: 54–59. doi:10.1016/s1369-5274(99)00051-x**

**71: Lawley TD, Chan K, Thompson LJ, Kim CC, Govoni GR, Monack DM. Genome-wide screen for Salmonella genes required for long-term systemic infection of the mouse. PLoS Pathog. 2006;2: e11. doi:10.1371/journal.ppat.0020011**

**Morevover, to be more precise, “faster” has been replaced by “more important” and “iii) a more preponderant role of the intracellular cycle of *Salmonella* compared to *Shigella”* has been suppressed**

Finally, in my point of view this work deserves publishing in that esteemed journal.

Best,

**Thank you very much**

Reviewer #3: The manuscript is recommended for publication once the minor revision is addressed.

**We thank the reviewer for this recommendation.**

Reviewer #4: The goal of the manuscript was to examine the immunogenicity and protective efficacy of the homologous type-III secretion system tip proteins IpaD from Shigella flexneri and SipD from Salmonella following intranasal and intragastric administration in a mouse model. Using various ELISA assays to measure antibody responses as well as performing lethal challenge studies in mice, the authors were able to detect IgG and IgA-specific responses and demonstrated that mice immunized with the proteins protected against each pathogen. More importantly, the authors provide evidence that the proteins can provide cross-protection against the other pathogen, which has important implications for vaccine development. Throughout the study, the authors perform robust analyses with a significant number of mice per treatment group, in which mice were immunized on days 0, 21, and 42 and challenged studies were performed on day 84. Results of the antibody responses in both the figures and supplemental figures were provided for both endpoint titers and throughout the course of the immunizations. In all, the data provide promising results for the pre-clinical efficacy analysis of the antibodies generated and the protection of mice that results from immunization of IpaD and SipD proteins.

**We thank the reviewer for this comment.**

However, review of the manuscript identified some major and minor concerns that are highlighted below.

Major:

1. The authors state that the recombinant IpaD and SipD proteins were purified by cloning a poly-histidine tag on the 3’ end of the gene sequence. Moreover, the labeling of the recombinant proteins with biotin for the ELISAs appears to have the histidine tag intact for this procedure. There are no histidine-only controls provided in the analyses to determine if the antibodies generated are specific to the histidine tag. These controls are not only important for determining IpaD- or SipD-specific antibody responses, but are also important when evaluating the cross-protection analysis performed in the study. It is possible that the cross-protection is due to some or all the histidine tag present on the recombinant proteins and not due to the 38% identity between the two proteins.

**We have added a figure (supplementary figure S3) showing that there is no cross-reaction in the immune response that could be due to the His Tag. When the ELISA was performed, a control was usaully added, using an unrelated recombinant protein possessing the His tag at its C-terminus. As shown in Figure S3, we used PrgI (protein of the needle of the Salmonella injectisome) as control for a mouse immunized with SipD and MxiH (protein of the needle of the Shigella injectisome) as control for a mouse immunized with IpaD. These controls were not performed for each mouse (to avoid the manipulation of huge amounts of ELISA but each time it was done, we did not see any cross-reactivity. A sentence in the experimental procedure has been added to precise this point (line 207 « Unrelated biotinylated recombinant proteins sharing also an His-tag at their C-terminus were sometimes added as controls (PrgI for SipD immunized mice and MxiH for IpaD immunized mice »).**

**I would like to add that my lab is performing a lot of immunizations of mice for the production of monoclonal antibodies, and each time we performed this type of controls for other recombinant proteins not related to this subject, we never observed cross-reactivity due to the His tag.**

2. The authors utilize IgG ratio analyses (Figure 4) to determine if the results are indicative of a humoral or cellular immune response of T helper cells. Direct measures and identification of specific T cell responses were not performed, which would strengthen the data. In the absence of such analyses, the author should state any conclusion from the ratio analyses as a hypothesis.

**We agree with the reviewer and as no T cell response has been measured, it remains only an hypothesis. A sentence has been added in the result section line 323 « However this result should be taken with caution as it has not been confirmed by measuring directly the T cell specific response »** **as well as a pragraph in the discussion line 495 « All antibody subclasses are involved in the humoral response for both SipD and IpaD, regardless of route of immunization, and this highlights the importance of humoral (and particularly mucosal) immunity in protecting against *Salmonella* and *Shigella* infection. It has to be noted that protection against *Shigella* infection is better when mice are immunized by the IG route compared to the IN route. This might be correlated to the IgG subtype measurement balance in favor of a cellular response for IpaD by the IG route suggesting a significant contribution of the Th1 response for protection after IN challenge. It has to be noted that this hypothesis has not been verified by a direct measurement of the T cell specific response.»**

2. While the challenge studies use 100 LD50, there is concern that the LD50 dose used by the authors for S. flexneri resulted in 80% survival and not 50% survival (see supplemental figure 5). As the authors point out, the challenge dose of 5.1 x 10^10 is very high, but justify this high dose by pointing to a Shigella study in 2012 in which high LD50s were also used. This discussion is misleading, especially since the referenced study used doses of 6 x 10^7 and 1.3 x 10^8 for S. flexneri. There is a significant concern that the protection observed in the manuscript is not as promising given the dosages and model used. Furthermore, the concern is raised even more given the fact that the infection model is pulmonary-based and not physiologically relevant to Shigella infection. Animal models for Shigella can be difficult and vaccine development has not been successful despite promising animal model analyses throughout the literature. In the absence of repeating the analyses with better infection results as performed by other studies, the authors should better highlight and discuss the shortcomings from their analyses.

**It has to be noted that there is probably a confusion between number of cfu/mL and number of cfu/mouse. 100 LD50 in our study corresponds to 5X10^10^ cfu/mL and 10^9^ cfu/mouse (administration of 20 µL of the solution at 5X10^10^ cfu/ml). It has been precised in the manuscript (lines 372-373).**

**The LD50 of *S. flexneri 2a* has been calculated (reed and Muench method) at** **10^7^ cfu/mouse (5.10^8^ cfu/mL), and we agree that, at this concentration, we obtained 80% mice survival instead of 50%. However this dose is in agreement with the literature (Van de Verg LL, et al, Infection and Immunity, 1995) and 10 fold superior to what was obtained by Phalipon et al, 1995, who gave us the strain.**

**Moreover, we have cited the publication of Martinez-Becerra FJ et al, 2012, where they determined the LD50 at 5.4 × 10^6^ CFU/mouse for *S. flexneri* and 1.2 × 10^7^ CFU/mouse for *S. sonnei* by the method of Reed and Muench, which is very close to our LD50 (10^7^ cfu/mouse). The highest dose used in this study, was 24 LD50 for S. flexneri = 1.3 10^8^ cfu/mouse, compared to 100 LD50 (10^9^ cfu/mouse) in our study.**

**It is well-known that the mouse model for shigellosis is inadequate and that pulmonary-based model is not relevant for colonization of the intestinal tract. However, although far from perfect, the pulmonary based mouse model for shigellosis is the one used by the scientific community working on shigella vaccine.**

**We still think that our study does not demerit in comparison to other studies and especially this study of 2012 : the challenge in our study was higher (with comparable determined LD50) using the same model (intranasal infection).**

**We have added a sentence, line 458 in the discussion about the pulmonary model of Shigella infection :**

**« Although it is recognized that the mouse model of *Shigella* pulmonary infection is not ideal to mimick an intestinal infection, it is currently the one used by scientific community for the evaluation of vaccines in development. »**

Minor:

1. The figure legends should define what the controls were for the analyses. It is assumed that the controls were mice treated with adjuvant + PBS, but the clarity would be helpful. It should also be stated in the Materials and Methods how many mice were used for the control groups. 15 is assumed but clarification would be helpful.

**It has been corrected in the legends. We used mice immunized intranasally with PBS+adjuvant for the controls, and the number was the same (15 mice per group). It has been added in the experimental procedure as well (line 181)**

2. The figure legends should better clarify the immunogen and recombinant protein sentences (e.g. for Fig 1, lines 216-217). Suggestion is “…indicates biotinylated recombinant protein used for the ELISA analyses.”

**All the legends have been corrected.**

3. The previous publication by the group in 2016 examined the secondary structure of recombinant SipD and PrgI using Far-UV CD spectroscopy. It is not clear why this analysis was not performed for IpaD.

**Regarding the circular dichroism (CD), it has been performed for SipD for the first article of B. Jneid (B. Jneid et al, 2016) and at this time IpaD was not produced. It has not been done after. We don’t have any more protein that was used for the immunizations. Thus, it does not seem relevant to reproduce now the protein to perform the CD, as it won’t be the same batch.**

**RESPONSES TO REVIEWERS (attached word document of the mail)**

**We thank reviewers for having reviewed carefully our publication and for all their comments, which allowed us to improve the manuscript. We have answered the best we could to the different points. We hope that reviewers will find it satisfactory for publication.**

Dear Editor,

I read and enjoyed the present work. The main Idea was novel, rationale and interesting, experiments were designed appropriately and the results were presented and discussed professionally. I congratulate the authors for doing this study. However, there were some minor points that I'll mention in the following.

**I gratefully thank the reviewer for its very positive comment and will do my best to answer all the mentioned points carefully.**

Since the authors purified the recombinant proteins, I think it was better if they share some photos of the recombinant proteins.

**I am sorry if I don’t understand well the question. We already have shown the SDS PAGE electrophoresis in supplementary S1 figure. Except that, we do not have the possibility to perform any electron microscopy. Regarding the circular dichroism (CD), it has been performed for SipD for the first article of B. Jneid (B. Jneid et al, 2016) and at this time IpaD was not produced. It has not been done after. We don’t have any more protein that was used for the immunizations. Thus, it does not seem relevant to reproduce now the protein to perform the CD, as it won’t be the same batch.**

The authors have done IgG isotyping, however, they did not discuss the results of this experiment in "Discussion" part.

**We have added a pragraph in the discussion line 495 « All antibody subclasses are involved in the humoral response for both SipD and IpaD, regardless of route of immunization, and this highlights the importance of humoral (and particularly mucosal) immunity in protecting against Salmonella and Shigella infection. It has to be noted that protection against Shigella infection is better when mice are immunized by the IG route compared to the IN route. This might be correlated to the IgG subtype measurement balance in favor of a cellular response for IpaD by the IG route suggesting a significant contribution of the Th1 response for protection after IN challenge. It has to be noted that this hypothesis has not been verified by a direct measurement of the T cell specific response.»**

Somewhere in the manuscript, mainly from line 394 to the end of the "Discussion", no reference has mentioned, though it is necessary to refer to the previous studies. For example, i) Salmonella has two type three secretion systems involved in the pathogenicity [Reference is needed], ii) pathogenicity mechanisms are different between Salmonella and Shigella and particularly in regard to the involvement of the innate and adaptative immune response, iii) a more preponderant role of the intracellular cycle of Salmonella compared to Shigella [Reference is needed] and iv) a faster systemic dissemination of Salmonella in the murine model [Reference is needed]...

**References have been inserted to support these hypotheses: for i) ref 69 has been added, for iii) ref 70 and 71 have been added.**

**69 : Silva-Herzog E, Detweiler CS. Salmonella enterica replication in hemophagocytic macrophages requires two type three secretion systems. Infect Immun. 2010;78: 3369–3377. doi:10.1128/IAI.00292-10**

**70: Vazquez-Torres A, Fang FC. Cellular routes of invasion by enteropathogens. Curr Opin Microbiol. 2000;3: 54–59. doi:10.1016/s1369-5274(99)00051-x**

**71: Lawley TD, Chan K, Thompson LJ, Kim CC, Govoni GR, Monack DM. Genome-wide screen for Salmonella genes required for long-term systemic infection of the mouse. PLoS Pathog. 2006;2: e11. doi:10.1371/journal.ppat.0020011**

**Morevover, to be more precise, “faster” has been replaced by “more important” and “iii) a more preponderant role of the intracellular cycle of *Salmonella* compared to *Shigella”* has been suppressed**

Furthermore, there were some little writing mistakes that I should mention to be corrected:

-In the first line of the abstract please write food- and water-borne pathogens

**It has been corrected**

-In line 37, please write emergence of multi drug resistances.

**Corrected. “new” has been removed.**

-In the lane 74, what is 30?

**This was a mistake of bibliography formatting. It has been corrected and the corresponding reference has been added:**

**Galan JE (2001) Salmonella interactions with host cells: type III secretion at work. Annu. Rev. Cell Dev. Biol. 17: 53–86**

-Please italicize family, genus, species and subspecies all over the manuscript. (Line 48, for example).

**It has been corrected for *Shigella flexneri*. According to the nomenclature (which has evolved with time), *Salmonella enterica* subsp *enterica* serovar Typhimurium should be italicized for *Salmonella* and *enterica*, but should be written as it (Typhimurium, not italicized, with T in capital). To clarify: 1- in the abstract (lines 23-24) “*Salmonella* Typhimurium” has been replaced by “*Salmonella* *enterica* serovar Typhimurium (*S.* Typhimurium)”, 2- line 29 *Salmonella* Typhimurium has been replaced by *S.* Typhimurium, 3- lines 65-66 S. Typhi has been replaced by *S. enterica* serovar Typhi.**

-Please capitalize the word "Gram" in line 79.

**Corrected, lines 49 and 87**

-In Lane 135, please write "phosphate-buffered saline".

**corrected**

-I think there should be a space between the numbers (4, 37, etc.) and ºC. Please correct it all over the text.

**corrected**

-In line 142, there should be a space between g and for.

**corrected**

-In line 143 and 144, please write 2 × 102 to 2 × 108	CFU and 5 × 105 to 5 × 1010	CFU.

**corrected**

-All over the text you have used capital L for showing the volume, but somewhere, mainly in "Enzyme immunoassays" part in Materials and Methods section. Please correct this.

**Corrected all along the text**

-In line 167, there should be a space between 10 and μg/mL.

**corrected**

Finally, in my point of view this work deserves publishing in that esteemed journal.

**Thank you very much for this very positive comment.**

Best,

This is a well written manuscript giving a reasoned account of how the authors selected the antigens to induce a cross-protection against Shigella and Salmonella infections in a mouse model. The experiment was well designed and the homologous and heterologous antibody responses following the selected antigens immunization makes this work interesting.

**We are grateful to the reviewer for its positive comments.**

1. There are minor scientific language deficiencies in the manuscript. 2. Some of the scientific terms such as “S. typhi”, “S. typhimurium” and “Shigella flexneri” are not italized.

**It has been corrected for *Shigella flexneri*. According to the nomenclature (which has evolved with time), *Salmonella enterica* subsp *enterica* serovar Typhimurium should be italicized for *Salmonella* and *enterica*, but should be written as it (Typhimurium, not italicized, with T in capital). To clarify: 1- in the abstract (lines 23-24) “*Salmonella* Typhimurium” has been replaced by “*Salmonella* *enterica* serovar Typhimurium (*S.* Typhimurium)”, 2- line 29 *Salmonella* Typhimurium has been replaced by *S.* Typhimurium, 3- lines 65-66 S. Typhi has been replaced by *S. enterica* serovar Typhi.**

3. Capitalize Gram-negative.

**Corrected, lines 49 and 87**

4. Line 142: 2,000 x g for… (space) : **corrected**

5. Table 1: The numbering shown in the table sounds confusing. Do they mean A x B y (e.g. 1.7 x 103 )?

**The reviewer is right, it has been corrected.**

6. Can author provide additional explanation why were only SipD and IpaD selected in this work given Salmonella and Shigella have more relevant proteins (e.g. IpaA and IpaC for Shigella).

**Regarding Salmonella, a first study we have performed some years ago provided encouraging results with SipD (Jneid B, et al., 2016). As numerous publications also focused on IpaD for Shigella, we decided to start this comparative study, assuming that the mechanism was probably the same between Salmonella and Shigella and that epitopes involved in protection might be the same.**

7. Line 43: 38% sequence identity between Salmonella and Shigella. Please provide exact species of organisms used for sequence comparison. A BLAST result of the sequence comparison is required for this manuscript.

**As indicated in Supp Fig 7, a BLAST has been performed between IpaD of *Shigella flexneri 2a* (accession number SVF87366.1) and SipD of *S.* Typhimurium (accession number AAA86617.1). The BLAST gave a sequence identity of 38.17%. The sentence “Sequence identity is 38.17%.” has been added in the legend of Supp Fig 7. Please refer also to line 353.**

8. Did author express whole protein or truncated protein of the selected antigens? Please provide the primers used for cloning.

**The whole proteins have been expressed. Line 136: a table showing the sequences of the primers used has been inserted. Line 139: “Whole proteins” have been added**

9. Figures 1 – 6 are not clear. It is recommended the authors could provide a better resolution of the figures.

**We have increased the quality of the figures. I hope that it will appear satisfactory in the pdf version sent for the revision.**

 
We cannot make any decision about publication until we have seen the revised manuscript and your response to the reviewers' comments. Your revised manuscript is also likely to be sent to reviewers for further evaluation.
 
When you are ready to resubmit, please upload the following:

[1] A letter containing a detailed list of your responses to the review comments and a description of the changes you have made in the manuscript. Please note while forming your response, if your article is accepted, you may have the opportunity to make the peer review history publicly available. The record will include editor decision letters (with reviews) and your responses to reviewer comments. If eligible, we will contact you to opt in or out.

[2] Two versions of the revised manuscript: one with either highlights or tracked changes denoting where the text has been changed; the other a clean version (uploaded as the manuscript file).

Important additional instructions are given below your reviewer comments.

Please prepare and submit your revised manuscript within 60 days. If you anticipate any delay, please let us know the expected resubmission date by replying to this email. Please note that revised manuscripts received after the 60-day due date may require evaluation and peer review similar to newly submitted manuscripts.
 
Thank you again for your submission. We hope that our editorial process has been constructive so far, and we welcome your feedback at any time. Please don't hesitate to contact us if you have any questions or comments.
 
Sincerely,

Alfredo G Torres
Deputy Editor
PLOS Neglected Tropical Diseases

Alfredo Torres
Deputy Editor
PLOS Neglected Tropical Diseases

***********************


Reviewer's Responses to Questions

**Key Review Criteria Required for Acceptance?**


As you describe the new analyses required for acceptance, please consider the following:


**Methods**


-Are the objectives of the study clearly articulated with a clear testable hypothesis stated?

-Is the study design appropriate to address the stated objectives?

-Is the population clearly described and appropriate for the hypothesis being tested?

-Is the sample size sufficient to ensure adequate power to address the hypothesis being tested?

-Were correct statistical analysis used to support conclusions?

-Are there concerns about ethical or regulatory requirements being met?


Reviewer #1: The methods that are described are sufficient but it is unclear why the authors chose to measure Ig concentrations in ng/ml rather than the typical titers. Also, no discussion of PrgI production is presented making that protein irrelevant.
The LD50 for Shigella is very, very high. There is no discussion of differential colony staining on Congo red. It is likely that the vast majority of colonies are white producing an artificially high LD50. At these challenge doses all the mice should be dead in 24 hr.

Reviewer #2: Methods have been done and presented properly.

Reviewer #3: This is a well written manuscript giving a reasoned account of how the authors selected the antigens to induce a cross-protection against Shigella and Salmonella infections in a mouse model. The experiment was well designed and the homologous and heterologous antibody responses following the selected antigens immunization makes this work interesting.

Reviewer #4: As seen in the comments to the authors, the following concerns are noted for the methods:
- the analyses used histidine-tagged proteins or biotinylated the tagged proteins for the ELISA analyses
- no direct measure for T cell-specific responses.
- the LD50 for the Shigella infection resulted in an 80% survival and required a very high infectious dose for the challenge studies.

--------------------

**Results**


-Does the analysis presented match the analysis plan?

-Are the results clearly and completely presented?

-Are the figures (Tables, Images) of sufficient quality for clarity?


Reviewer #1: The results are presented but for most of the manuscript are overstated. It is hard to the manuscript seriously when IpaD does not provide protection after IN vaccination and IN challenge. That should have been the positive control. Again, with such a high dose of Shigella, it is unclear how any of the mice could have survived. Like the shigella are "white" and are missing the T3SS.
The authors need to decide on OG vs IG. This is OG ORO-gastric

Reviewer #2: Like Methods, results have been done and presented properly. However, since the authors purified the recombinant proteins, I think it was better if they share some photos of the recombinant proteins.

Reviewer #3: The results are completely presented. However, the quality and resolution for Figures 1- 6 are not satisfied.

Reviewer #4: The analyses match the plan and the figures/tables are presented clearly. Please note the minor concerns to improve the clarity of the figure legends.

--------------------

**Conclusions**


-Are the conclusions supported by the data presented?

-Are the limitations of analysis clearly described?

-Do the authors discuss how these data can be helpful to advance our understanding of the topic under study?

-Is public health relevance addressed?


Reviewer #1: The conclusions are NOT supported by the results. Many of the conclusions drawn by the authors lack significance - especially in the challenge experiments.

Reviewer #2: (No Response)

Reviewer #3: Conclusion is well-defined and supports the data presented. It is helpful to advance our understanding of the topic under study.

Reviewer #4: Based on the concerns noted in the methods and in the comments to the authors, some of the conclusions are not supported by the data presented. In particular, the cross-protection, efficacy in the Shigella challenge model, and the T helper cellular responses are not supported. The limitations to these are not clearly described.

--------------------

**Editorial and Data Presentation Modifications?**


Use this section for editorial suggestions as well as relatively minor modifications of existing data that would enhance clarity. If the only modifications needed are minor and/or editorial, you may wish to recommend “Minor Revision” or “Accept”.

Reviewer #1: (No Response)

Reviewer #2: There were some little writing mistakes that I should mention to be corrected:
-In the first line of the abstract please write food- and water-borne pathogens
-In line 37, please write emergence of multi drug resistances.
-In the lane 74, what is 30?
-Please italicize family, genus, species and subspecies all over the manuscript. (Line 48, for example).
-Please capitalize the word "Gram" in line 79.
-In Lane 135, please write "phosphate-buffered saline".
-I think there should be a space between the numbers (4, 37, etc.) and ºC. Please correct it all over the text.
-In line 142, there should be a space between g and for.
-In line 143 and 144, please write 2 × 102 to 2 × 108 CFU and 5 × 105 to 5 × 1010 CFU.
-All over the text you have used capital L for showing the volume, but somewhere, mainly in "Enzyme immunoassays" part in Materials and Methods section. Please correct this.
-In line 167, there should be a space between 10 and μg/mL.

Reviewer #3: The manuscript is recommended for publication once the minor revision is addressed.

Reviewer #4: (No Response)

--------------------

**Summary and General Comments**


Use this section to provide overall comments, discuss strengths/weaknesses of the study, novelty, significance, general execution and scholarship. You may also include additional comments for the author, including concerns about dual publication, research ethics, or publication ethics. If requesting major revision, please articulate the new experiments that are needed.

Reviewer #1: (No Response)

Reviewer #2: I read and enjoyed the present work. The main Idea was novel, rationale and interesting, experiments were designed appropriately and the results were presented and discussed professionally. I congratulate the authors for doing this study. However, there were some minor points that I'll mention in the following.
1. Since the authors purified the recombinant proteins, I think it was better if they share some photos of the recombinant proteins.
2. The authors have done IgG isotyping, however, they did not discuss the results of this experiment in "Discussion" part.
3. Somewhere in the manuscript, mainly from line 394 to the end of the "Discussion", no reference has mentioned, though it is necessary to refer to the previous studies. For example, i) Salmonella has two type three secretion systems involved in the pathogenicity [Reference is needed], ii) pathogenicity mechanisms are different between Salmonella and Shigella and particularly in regard to the involvement of the innate and adaptative immune response, iii) a more preponderant role of the intracellular cycle of Salmonella compared to Shigella [Reference is needed] and iv) a faster systemic dissemination of Salmonella in the murine model [Reference is needed]...
Finally, in my point of view this work deserves publishing in that esteemed journal.
Best,

Reviewer #3: The manuscript is recommended for publication once the minor revision is addressed.

Reviewer #4: The goal of the manuscript was to examine the immunogenicity and protective efficacy of the homologous type-III secretion system tip proteins IpaD from Shigella flexneri and SipD from Salmonella following intranasal and intragastric administration in a mouse model. Using various ELISA assays to measure antibody responses as well as performing lethal challenge studies in mice, the authors were able to detect IgG and IgA-specific responses and demonstrated that mice immunized with the proteins protected against each pathogen. More importantly, the authors provide evidence that the proteins can provide cross-protection against the other pathogen, which has important implications for vaccine development. Throughout the study, the authors perform robust analyses with a significant number of mice per treatment group, in which mice were immunized on days 0, 21, and 42 and challenged studies were performed on day 84. Results of the antibody responses in both the figures and supplemental figures were provided for both endpoint titers and throughout the course of the immunizations. In all, the data provide promising results for the pre-clinical efficacy analysis of the antibodies generated and the protection of mice that results from immunization of IpaD and SipD proteins. However, review of the manuscript identified some major and minor concerns that are highlighted below.

Major:
1. The authors state that the recombinant IpaD and SipD proteins were purified by cloning a poly-histidine tag on the 3’ end of the gene sequence. Moreover, the labeling of the recombinant proteins with biotin for the ELISAs appears to have the histidine tag intact for this procedure. There are no histidine-only controls provided in the analyses to determine if the antibodies generated are specific to the histidine tag. These controls are not only important for determining IpaD- or SipD-specific antibody responses, but are also important when evaluating the cross-protection analysis performed in the study. It is possible that the cross-protection is due to some or all the histidine tag present on the recombinant proteins and not due to the 38% identity between the two proteins.

2. The authors utilize IgG ratio analyses (Figure 4) to determine if the results are indicative of a humoral or cellular immune response of T helper cells. Direct measures and identification of specific T cell responses were not performed, which would strengthen the data. In the absence of such analyses, the author should state any conclusion from the ratio analyses as a hypothesis.

3. While the challenge studies use 100 LD50, there is concern that the LD50 dose used by the authors for S. flexneri resulted in 80% survival and not 50% survival (see supplemental figure 5). As the authors point out, the challenge dose of 5.1 x 10^10 is very high, but justify this high dose by pointing to a Shigella study in 2012 in which high LD50s were also used. This discussion is misleading, especially since the referenced study used doses of 6 x 10^7 and 1.3 x 10^8 for S. flexneri. There is a significant concern that the protection observed in the manuscript is not as promising given the dosages and model used. Furthermore, the concern is raised even more given the fact that the infection model is pulmonary-based and not physiologically relevant to Shigella infection. Animal models for Shigella can be difficult and vaccine development has not been successful despite promising animal model analyses throughout the literature. In the absence of repeating the analyses with better infection results as performed by other studies, the authors should better highlight and discuss the shortcomings from their analyses.


Minor:
1. The figure legends should define what the controls were for the analyses. It is assumed that the controls were mice treated with adjuvant + PBS, but the clarity would be helpful. It should also be stated in the Materials and Methods how many mice were used for the control groups. 15 is assumed but clarification would be helpful.

2. The figure legends should better clarify the immunogen and recombinant protein sentences (e.g. for Fig 1, lines 216-217). Suggestion is “…indicates biotinylated recombinant protein used for the ELISA analyses.”

3. The previous publication by the group in 2016 examined the secondary structure of recombinant SipD and PrgI using Far-UV CD spectroscopy. It is not clear why this analysis was not performed for IpaD.Reviewer #1: The methods that are described are sufficient but it is unclear why the authors chose to measure Ig concentrations in ng/ml rather than the typical titers. Also, no discussion of PrgI production is presented making that protein irrelevant.
The LD50 for Shigella is very, very high. There is no discussion of differential colony staining on Congo red. It is likely that the vast majority of colonies are white producing an artificially high LD50. At these challenge doses all the mice should be dead in 24 hr.

Reviewer #2: Methods have been done and presented properly.

Reviewer #3: This is a well written manuscript giving a reasoned account of how the authors selected the antigens to induce a cross-protection against Shigella and Salmonella infections in a mouse model. The experiment was well designed and the homologous and heterologous antibody responses following the selected antigens immunization makes this work interesting.

Reviewer #4: As seen in the comments to the authors, the following concerns are noted for the methods:
- the analyses used histidine-tagged proteins or biotinylated the tagged proteins for the ELISA analyses
- no direct measure for T cell-specific responses.
- the LD50 for the Shigella infection resulted in an 80% survival and required a very high infectious dose for the challenge studies.

--------------------

**Results**


-Does the analysis presented match the analysis plan?

-Are the results clearly and completely presented?

-Are the figures (Tables, Images) of sufficient quality for clarity?


Reviewer #1: The results are presented but for most of the manuscript are overstated. It is hard to the manuscript seriously when IpaD does not provide protection after IN vaccination and IN challenge. That should have been the positive control. Again, with such a high dose of Shigella, it is unclear how any of the mice could have survived. Like the shigella are "white" and are missing the T3SS.
The authors need to decide on OG vs IG. This is OG ORO-gastric

Reviewer #2: Like Methods, results have been done and presented properly. However, since the authors purified the recombinant proteins, I think it was better if they share some photos of the recombinant proteins.

Reviewer #3: The results are completely presented. However, the quality and resolution for Figures 1- 6 are not satisfied.

Reviewer #4: The analyses match the plan and the figures/tables are presented clearly. Please note the minor concerns to improve the clarity of the figure legends.

--------------------

**Conclusions**


-Are the conclusions supported by the data presented?

-Are the limitations of analysis clearly described?

-Do the authors discuss how these data can be helpful to advance our understanding of the topic under study?

-Is public health relevance addressed?


Reviewer #1: The conclusions are NOT supported by the results. Many of the conclusions drawn by the authors lack significance - especially in the challenge experiments.

Reviewer #2: (No Response)

Reviewer #3: Conclusion is well-defined and supports the data presented. It is helpful to advance our understanding of the topic under study.

Reviewer #4: Based on the concerns noted in the methods and in the comments to the authors, some of the conclusions are not supported by the data presented. In particular, the cross-protection, efficacy in the Shigella challenge model, and the T helper cellular responses are not supported. The limitations to these are not clearly described.

--------------------

**Editorial and Data Presentation Modifications?**


Use this section for editorial suggestions as well as relatively minor modifications of existing data that would enhance clarity. If the only modifications needed are minor and/or editorial, you may wish to recommend “Minor Revision” or “Accept”.

Reviewer #1: (No Response)

Reviewer #2: There were some little writing mistakes that I should mention to be corrected:
-In the first line of the abstract please write food- and water-borne pathogens
-In line 37, please write emergence of multi drug resistances.
-In the lane 74, what is 30?
-Please italicize family, genus, species and subspecies all over the manuscript. (Line 48, for example).
-Please capitalize the word "Gram" in line 79.
-In Lane 135, please write "phosphate-buffered saline".
-I think there should be a space between the numbers (4, 37, etc.) and ºC. Please correct it all over the text.
-In line 142, there should be a space between g and for.
-In line 143 and 144, please write 2 × 102 to 2 × 108 CFU and 5 × 105 to 5 × 1010 CFU.
-All over the text you have used capital L for showing the volume, but somewhere, mainly in "Enzyme immunoassays" part in Materials and Methods section. Please correct this.
-In line 167, there should be a space between 10 and μg/mL.

Reviewer #3: The manuscript is recommended for publication once the minor revision is addressed.

Reviewer #4: (No Response)

--------------------

**Summary and General Comments**


Use this section to provide overall comments, discuss strengths/weaknesses of the study, novelty, significance, general execution and scholarship. You may also include additional comments for the author, including concerns about dual publication, research ethics, or publication ethics. If requesting major revision, please articulate the new experiments that are needed.

Reviewer #1: (No Response)

Reviewer #2: I read and enjoyed the present work. The main Idea was novel, rationale and interesting, experiments were designed appropriately and the results were presented and discussed professionally. I congratulate the authors for doing this study. However, there were some minor points that I'll mention in the following.
1. Since the authors purified the recombinant proteins, I think it was better if they share some photos of the recombinant proteins.
2. The authors have done IgG isotyping, however, they did not discuss the results of this experiment in "Discussion" part.
3. Somewhere in the manuscript, mainly from line 394 to the end of the "Discussion", no reference has mentioned, though it is necessary to refer to the previous studies. For example, i) Salmonella has two type three secretion systems involved in the pathogenicity [Reference is needed], ii) pathogenicity mechanisms are different between Salmonella and Shigella and particularly in regard to the involvement of the innate and adaptative immune response, iii) a more preponderant role of the intracellular cycle of Salmonella compared to Shigella [Reference is needed] and iv) a faster systemic dissemination of Salmonella in the murine model [Reference is needed]...
Finally, in my point of view this work deserves publishing in that esteemed journal.
Best,

Reviewer #3: The manuscript is recommended for publication once the minor revision is addressed.

Reviewer #4: The goal of the manuscript was to examine the immunogenicity and protective efficacy of the homologous type-III secretion system tip proteins IpaD from Shigella flexneri and SipD from Salmonella following intranasal and intragastric administration in a mouse model. Using various ELISA assays to measure antibody responses as well as performing lethal challenge studies in mice, the authors were able to detect IgG and IgA-specific responses and demonstrated that mice immunized with the proteins protected against each pathogen. More importantly, the authors provide evidence that the proteins can provide cross-protection against the other pathogen, which has important implications for vaccine development. Throughout the study, the authors perform robust analyses with a significant number of mice per treatment group, in which mice were immunized on days 0, 21, and 42 and challenged studies were performed on day 84. Results of the antibody responses in both the figures and supplemental figures were provided for both endpoint titers and throughout the course of the immunizations. In all, the data provide promising results for the pre-clinical efficacy analysis of the antibodies generated and the protection of mice that results from immunization of IpaD and SipD proteins. However, review of the manuscript identified some major and minor concerns that are highlighted below.

Major:
1. The authors state that the recombinant IpaD and SipD proteins were purified by cloning a poly-histidine tag on the 3’ end of the gene sequence. Moreover, the labeling of the recombinant proteins with biotin for the ELISAs appears to have the histidine tag intact for this procedure. There are no histidine-only controls provided in the analyses to determine if the antibodies generated are specific to the histidine tag. These controls are not only important for determining IpaD- or SipD-specific antibody responses, but are also important when evaluating the cross-protection analysis performed in the study. It is possible that the cross-protection is due to some or all the histidine tag present on the recombinant proteins and not due to the 38% identity between the two proteins.

2. The authors utilize IgG ratio analyses (Figure 4) to determine if the results are indicative of a humoral or cellular immune response of T helper cells. Direct measures and identification of specific T cell responses were not performed, which would strengthen the data. In the absence of such analyses, the author should state any conclusion from the ratio analyses as a hypothesis.

3. While the challenge studies use 100 LD50, there is concern that the LD50 dose used by the authors for S. flexneri resulted in 80% survival and not 50% survival (see supplemental figure 5). As the authors point out, the challenge dose of 5.1 x 10^10 is very high, but justify this high dose by pointing to a Shigella study in 2012 in which high LD50s were also used. This discussion is misleading, especially since the referenced study used doses of 6 x 10^7 and 1.3 x 10^8 for S. flexneri. There is a significant concern that the protection observed in the manuscript is not as promising given the dosages and model used. Furthermore, the concern is raised even more given the fact that the infection model is pulmonary-based and not physiologically relevant to Shigella infection. Animal models for Shigella can be difficult and vaccine development has not been successful despite promising animal model analyses throughout the literature. In the absence of repeating the analyses with better infection results as performed by other studies, the authors should better highlight and discuss the shortcomings from their analyses.


Minor:
1. The figure legends should define what the controls were for the analyses. It is assumed that the controls were mice treated with adjuvant + PBS, but the clarity would be helpful. It should also be stated in the Materials and Methods how many mice were used for the control groups. 15 is assumed but clarification would be helpful.

2. The figure legends should better clarify the immunogen and recombinant protein sentences (e.g. for Fig 1, lines 216-217). Suggestion is “…indicates biotinylated recombinant protein used for the ELISA analyses.”

3. The previous publication by the group in 2016 examined the secondary structure of recombinant SipD and PrgI using Far-UV CD spectroscopy. It is not clear why this analysis was not performed for IpaD. Reviewer #1: The methods that are described are sufficient but it is unclear why the authors chose to measure Ig concentrations in ng/ml rather than the typical titers. Also, no discussion of PrgI production is presented making that protein irrelevant.
The LD50 for Shigella is very, very high. There is no discussion of differential colony staining on Congo red. It is likely that the vast majority of colonies are white producing an artificially high LD50. At these challenge doses all the mice should be dead in 24 hr.

Reviewer #2: Methods have been done and presented properly.

Reviewer #3: This is a well written manuscript giving a reasoned account of how the authors selected the antigens to induce a cross-protection against Shigella and Salmonella infections in a mouse model. The experiment was well designed and the homologous and heterologous antibody responses following the selected antigens immunization makes this work interesting.

Reviewer #4: As seen in the comments to the authors, the following concerns are noted for the methods:
- the analyses used histidine-tagged proteins or biotinylated the tagged proteins for the ELISA analyses
- no direct measure for T cell-specific responses.
- the LD50 for the Shigella infection resulted in an 80% survival and required a very high infectious dose for the challenge studies.

--------------------

**Results**


-Does the analysis presented match the analysis plan?

-Are the results clearly and completely presented?

-Are the figures (Tables, Images) of sufficient quality for clarity?


Reviewer #1: The results are presented but for most of the manuscript are overstated. It is hard to the manuscript seriously when IpaD does not provide protection after IN vaccination and IN challenge. That should have been the positive control. Again, with such a high dose of Shigella, it is unclear how any of the mice could have survived. Like the shigella are "white" and are missing the T3SS.
The authors need to decide on OG vs IG. This is OG ORO-gastric

Reviewer #2: Like Methods, results have been done and presented properly. However, since the authors purified the recombinant proteins, I think it was better if they share some photos of the recombinant proteins.

Reviewer #3: The results are completely presented. However, the quality and resolution for Figures 1- 6 are not satisfied.

Reviewer #4: The analyses match the plan and the figures/tables are presented clearly. Please note the minor concerns to improve the clarity of the figure legends.

--------------------

**Conclusions**


-Are the conclusions supported by the data presented?

-Are the limitations of analysis clearly described?

-Do the authors discuss how these data can be helpful to advance our understanding of the topic under study?

-Is public health relevance addressed?


Reviewer #1: The conclusions are NOT supported by the results. Many of the conclusions drawn by the authors lack significance - especially in the challenge experiments.

Reviewer #2: (No Response)

Reviewer #3: Conclusion is well-defined and supports the data presented. It is helpful to advance our understanding of the topic under study.

Reviewer #4: Based on the concerns noted in the methods and in the comments to the authors, some of the conclusions are not supported by the data presented. In particular, the cross-protection, efficacy in the Shigella challenge model, and the T helper cellular responses are not supported. The limitations to these are not clearly described.

--------------------

**Editorial and Data Presentation Modifications?**


Use this section for editorial suggestions as well as relatively minor modifications of existing data that would enhance clarity. If the only modifications needed are minor and/or editorial, you may wish to recommend “Minor Revision” or “Accept”.

Reviewer #1: (No Response)

Reviewer #2: There were some little writing mistakes that I should mention to be corrected:
-In the first line of the abstract please write food- and water-borne pathogens
-In line 37, please write emergence of multi drug resistances.
-In the lane 74, what is 30?
-Please italicize family, genus, species and subspecies all over the manuscript. (Line 48, for example).
-Please capitalize the word "Gram" in line 79.
-In Lane 135, please write "phosphate-buffered saline".
-I think there should be a space between the numbers (4, 37, etc.) and ºC. Please correct it all over the text.
-In line 142, there should be a space between g and for.
-In line 143 and 144, please write 2 × 102 to 2 × 108 CFU and 5 × 105 to 5 × 1010 CFU.
-All over the text you have used capital L for showing the volume, but somewhere, mainly in "Enzyme immunoassays" part in Materials and Methods section. Please correct this.
-In line 167, there should be a space between 10 and μg/mL.

Reviewer #3: The manuscript is recommended for publication once the minor revision is addressed.

Reviewer #4: (No Response)

--------------------

**Summary and General Comments**


Use this section to provide overall comments, discuss strengths/weaknesses of the study, novelty, significance, general execution and scholarship. You may also include additional comments for the author, including concerns about dual publication, research ethics, or publication ethics. If requesting major revision, please articulate the new experiments that are needed.

Reviewer #1: (No Response)

Reviewer #2: I read and enjoyed the present work. The main Idea was novel, rationale and interesting, experiments were designed appropriately and the results were presented and discussed professionally. I congratulate the authors for doing this study. However, there were some minor points that I'll mention in the following.
1. Since the authors purified the recombinant proteins, I think it was better if they share some photos of the recombinant proteins.
2. The authors have done IgG isotyping, however, they did not discuss the results of this experiment in "Discussion" part.
3. Somewhere in the manuscript, mainly from line 394 to the end of the "Discussion", no reference has mentioned, though it is necessary to refer to the previous studies. For example, i) Salmonella has two type three secretion systems involved in the pathogenicity [Reference is needed], ii) pathogenicity mechanisms are different between Salmonella and Shigella and particularly in regard to the involvement of the innate and adaptative immune response, iii) a more preponderant role of the intracellular cycle of Salmonella compared to Shigella [Reference is needed] and iv) a faster systemic dissemination of Salmonella in the murine model [Reference is needed]...
Finally, in my point of view this work deserves publishing in that esteemed journal.
Best,

Reviewer #3: The manuscript is recommended for publication once the minor revision is addressed.

Reviewer #4: The goal of the manuscript was to examine the immunogenicity and protective efficacy of the homologous type-III secretion system tip proteins IpaD from Shigella flexneri and SipD from Salmonella following intranasal and intragastric administration in a mouse model. Using various ELISA assays to measure antibody responses as well as performing lethal challenge studies in mice, the authors were able to detect IgG and IgA-specific responses and demonstrated that mice immunized with the proteins protected against each pathogen. More importantly, the authors provide evidence that the proteins can provide cross-protection against the other pathogen, which has important implications for vaccine development. Throughout the study, the authors perform robust analyses with a significant number of mice per treatment group, in which mice were immunized on days 0, 21, and 42 and challenged studies were performed on day 84. Results of the antibody responses in both the figures and supplemental figures were provided for both endpoint titers and throughout the course of the immunizations. In all, the data provide promising results for the pre-clinical efficacy analysis of the antibodies generated and the protection of mice that results from immunization of IpaD and SipD proteins. However, review of the manuscript identified some major and minor concerns that are highlighted below.

Major:
1. The authors state that the recombinant IpaD and SipD proteins were purified by cloning a poly-histidine tag on the 3’ end of the gene sequence. Moreover, the labeling of the recombinant proteins with biotin for the ELISAs appears to have the histidine tag intact for this procedure. There are no histidine-only controls provided in the analyses to determine if the antibodies generated are specific to the histidine tag. These controls are not only important for determining IpaD- or SipD-specific antibody responses, but are also important when evaluating the cross-protection analysis performed in the study. It is possible that the cross-protection is due to some or all the histidine tag present on the recombinant proteins and not due to the 38% identity between the two proteins.

2. The authors utilize IgG ratio analyses (Figure 4) to determine if the results are indicative of a humoral or cellular immune response of T helper cells. Direct measures and identification of specific T cell responses were not performed, which would strengthen the data. In the absence of such analyses, the author should state any conclusion from the ratio analyses as a hypothesis.

3. While the challenge studies use 100 LD50, there is concern that the LD50 dose used by the authors for S. flexneri resulted in 80% survival and not 50% survival (see supplemental figure 5). As the authors point out, the challenge dose of 5.1 x 10^10 is very high, but justify this high dose by pointing to a Shigella study in 2012 in which high LD50s were also used. This discussion is misleading, especially since the referenced study used doses of 6 x 10^7 and 1.3 x 10^8 for S. flexneri. There is a significant concern that the protection observed in the manuscript is not as promising given the dosages and model used. Furthermore, the concern is raised even more given the fact that the infection model is pulmonary-based and not physiologically relevant to Shigella infection. Animal models for Shigella can be difficult and vaccine development has not been successful despite promising animal model analyses throughout the literature. In the absence of repeating the analyses with better infection results as performed by other studies, the authors should better highlight and discuss the shortcomings from their analyses.


Minor:
1. The figure legends should define what the controls were for the analyses. It is assumed that the controls were mice treated with adjuvant + PBS, but the clarity would be helpful. It should also be stated in the Materials and Methods how many mice were used for the control groups. 15 is assumed but clarification would be helpful.

2. The figure legends should better clarify the immunogen and recombinant protein sentences (e.g. for Fig 1, lines 216-217). Suggestion is “…indicates biotinylated recombinant protein used for the ELISA analyses.”

3. The previous publication by the group in 2016 examined the secondary structure of recombinant SipD and PrgI using Far-UV CD spectroscopy. It is not clear why this analysis was not performed for IpaD.
