# Supplementary material for: SipD and IpaD induce a cross-protection against Shigella and Salmonella infections
Source: PLoS Negl Trop Dis. 2020 May 28;14(5):e0008326. doi: 10.1371/journal.pntd.0008326 (PMC7282677; doi:10.1371/journal.pntd.0008326)
Supplement: Attachment — Submitted filename: Reviewer Comments.docx [file pntd.0008326.s008.docx]

Dear Editor,

I read and enjoyed the present work. The main Idea was novel, rationale and interesting, experiments were designed appropriately and the results were presented and discussed professionally. I congratulate the authors for doing this study. However, there were some minor points that I'll mention in the following.

1. Since the authors purified the recombinant proteins, I think it was better if they share some photos of the recombinant proteins.
2. The authors have done IgG isotyping, however, they did not discuss the results of this experiment in "Discussion" part.
3. Somewhere in the manuscript, mainly from line 394 to the end of the "Discussion", no reference has mentioned, though it is necessary to refer to the previous studies. For example, i) Salmonella has two type three secretion systems involved in the pathogenicity [Reference is needed], ii) pathogenicity mechanisms are different between Salmonella and Shigella and particularly in regard to the involvement of the innate and adaptative immune response, iii) a more preponderant role of the intracellular cycle of Salmonella compared to Shigella [Reference is needed] and iv) a faster systemic dissemination of Salmonella in the murine model [Reference is needed]...

Furthermore, there were some little writing mistakes that I should mention to be corrected:

-In the first line of the abstract please write food- and water-borne pathogens

-In line 37, please write emergence of multi drug resistances.

-In the lane 74, what is ^30^?

-Please italicize family, genus, species and subspecies all over the manuscript. (Line 48, for example).

-Please capitalize the word "Gram" in line 79.

-In Lane 135, please write "phosphate-buffered saline".

-I think there should be a space between the numbers (4, 37, etc.) and ºC. Please correct it all over the text.

-In line 142, there should be a space between g and for.

-In line 143 and 144, please write 2 × 10^2^ to 2 × 10^8^	CFU and 5 × 10^5^ to 5 × 10^10^	CFU.

-All over the text you have used capital L for showing the volume, but somewhere, mainly in "Enzyme immunoassays" part in Materials and Methods section. Please correct this.

-In line 167, there should be a space between 10 and μg/mL.

Finally, in my point of view this work deserves publishing in that esteemed journal.

Best,
