# Supplementary material for: SipD and IpaD induce a cross-protection against Shigella and Salmonella infections
Source: PLoS Negl Trop Dis. 2020 May 28;14(5):e0008326. doi: 10.1371/journal.pntd.0008326 (PMC7282677; doi:10.1371/journal.pntd.0008326)
Supplement: Attachment — Submitted filename: Reviewer comment.pdf [file pntd.0008326.s009.pdf]

This is a well written manuscript giving a reasoned account of how the authors selected the antigens to induce a cross-protection against *Shigella* and *Salmonella* infections in a mouse model. The experiment was well designed and the homologous and heterologous antibody responses following the selected antigens immunization makes this work interesting.

1. There are minor scientific language deficiencies in the manuscript.
2. Some of the scientific terms such as "S. typhi", "S. typhimurium" and "Shigella flexneri" are not italized.
3. Capitalize Gram-negative.
4. Line 142: 2,000 *x g* for… (space
5. Table 1:The numbering shown in the table sounds confusing. Do they mean A x B^y(e.g. 1.7 x 10^3)?
6. Can author provide additional explanation why were only SipD and IpaD selected in this work given Salmonella and Shigella have more relevant proteins (e.g. IpaA and IpaC for Shigella).
7. Line 43: 38% sequence identity between Salmonella and Shigella. Please provide exact species of organisms used for sequence comparison. A BLAST result of the sequence comparison is required for this manuscript.
8. Did author express whole protein or truncated protein of the selected antigens? Please provide the primers used for cloning.
9. Figures 1 – 6 are not clear. It is recommended the authors could provide a better resolution of the figures.
